# Angiotensin-(1–7) Promotes Resolution of Eosinophilic Inflammation in an Experimental Model of Asthma

**DOI:** 10.3389/fimmu.2018.00058

**Published:** 2018-01-29

**Authors:** Giselle S. Magalhaes, Lívia C. Barroso, Alesandra C. Reis, Maria G. Rodrigues-Machado, Juliana F. Gregório, Daisy Motta-Santos, Aline C. Oliveira, Denise A. Perez, Lucíola S. Barcelos, Mauro M. Teixeira, Robson A. S. Santos, Vanessa Pinho, Maria Jose Campagnole-Santos

**Affiliations:** ^1^Department of Physiology and Biophysics, Biological Sciences Institute, Federal University of Minas Gerais, Belo Horizonte, Brazil; ^2^Department of Biochemistry and Immunology, Biological Sciences Institute, Federal University of Minas Gerais, Belo Horizonte, Brazil; ^3^Department of Morphology, Biological Sciences Institute, Federal University of Minas Gerais, Belo Horizonte, Brazil

**Keywords:** Mas receptor, apoptosis, efferocytosis, caspase 3, GATA3, NF-kB, lung remodeling, allergic lung inflammation

## Abstract

Defective apoptosis of eosinophils, the main leukocyte in the pathogenesis of asthma, and delay in its removal lead to lung damage and loss of pulmonary function due to failure in the resolution of inflammation. Here, we investigated the ability of angiotensin-(1–7) [Ang-(1–7)], a pivotal peptide of the renin–angiotensin system, to promote resolution of an allergic lung inflammatory response. Balb/c mice were sensitized and challenged with ovalbumin and treated with Ang-(1–7) at the peak of the inflammatory process. Bronchoalveolar lavage (BAL) fluid and lungs were collected 24 h after treatment. Different lung lobes were processed for histology to evaluate inflammatory cell infiltration, airway and pulmonary remodeling, total collagen staining, and measurements of (i) collagen I and III mRNA expression by qRT-PCR; (ii) ERK1/2, IκB-α, and GATA3 protein levels by Western blotting; and (iii) eosinophilic peroxidase activity. Total number of inflammatory cells, proportion of apoptotic eosinophils and immunofluorescence for caspase 3 and NF-κB in leukocytes were evaluated in the BAL. Mas receptor immunostaining was evaluated in mouse and human eosinophils. Engulfment of human polimorphonuclear cells by macrophages, efferocytosis, was evaluated *in vivo*. Ang-(1–7) reduced eosinophils in the lung and in the BAL, increased the number of apoptotic eosinophils, shown by histology criteria and by increase in caspase 3 immunostaining. Furthermore, Ang-(1–7) decreased NF-kB immunostaining in eosinophils, reduced GATA3, ERK1/2, and IκB-α expression in the lung and decreased pulmonary remodeling and collagen deposition. Importantly, Ang-(1–7) increased efferocytosis. Our results demonstrate, for the first time, Ang-(1–7) activates events that are crucial for resolution of the inflammatory process of asthma and promotion of the return of lung homeostasis, indicating Ang-(1–7) as novel endogenous inflammation-resolving mediator.

## Introduction

Resolution of inflammation is an active process that allows cessation of inflammation and re-establishment of tissue homeostasis (1–3). It is a process characterized by prevention of excessive trafficking of leukocytes to the site of damage, shutdown intracellular signaling molecules associated with cytokine production and leukocyte survival, induction of apoptosis of recruited inflammatory cells, and promotion of clearance of apoptotic leukocytes, i.e., efferocytosis (1–5).

In chronic inflammatory diseases, including asthma, failure to resolve the inflammatory process causes a persistent inflammation with consequent tissue destruction and loss of organ function (6). In this context, maintenance of inflammation alters pulmonary homeostasis and culminates with clinical manifestations that affect quality of life of asthmatic patients (7). It has been suggested that drugs that enhance resolution of inflammation can be useful for treatment of chronic inflammatory diseases, such as asthma (2, 4, 8).

Asthma is characterized by inflammation, pulmonary remodeling, and bronchial hyperresponsiveness, in which multiple cells and multiple mediators play a crucial pathophysiological role (7, 9–11). Inflammatory mediators that increase influx of leukocytes, activity, and survival of eosinophils are positively correlated with asthma severity (6, 12, 13). The large increase in incidence of asthma is becoming a major global health problem and has encouraged studies aimed at increasing the knowledge of the pathophysiology of asthma, as well as development of new treatments to improve clinical management of the disease, mainly to meet asthma patients who do not respond well to current therapies (14).

Angiotensin-(1–7) [Ang-(1–7)] is now recognized as an important mediator of the renin–angiotensin system (15). Ang-(1–7) can be synthesized in the circulation or in different tissues mainly by angiotensin-converting enzyme 2 (ACE2) (16, 17) and it exerts its actions largely through activation of Mas-G-protein-coupled receptor (18). ACE2/Ang-(1–7)/Mas receptor pathway has gained much interest in recent years because of its anti-inflammatory, anti-proliferative, and anti-fibrotic actions, which oppose to the well-known pro-inflammatory effects of the ACE/Ang II/AT_1_ receptor pathway (19).

Recently, we showed that Ang-(1–7) is reduced in the lung of animals with chronic allergic lung inflammation (20). In addition, we demonstrated that preventive administration of Ang-(1–7) or a synthetic analog, AVE0991, greatly attenuate lung inflammation and ameliorated pulmonary function in a model of chronic asthma induced by repeated exposure of immunized mice to ovalbumine (20, 21). Activation of ACE2/Ang-(1–7)/Mas axis has also been described to display anti-inflammatory action in other experimental models, including ischemic stroke (22), atherosclerosis (23), pulmonary fibrosis (24), acute lung injury (25), and arthritis (26). On the other hand, in Mas-deficient mice, inflammation was not only enhanced but also prolonged (26, 27), suggesting that resolution of inflammation may be altered in the absence/malfunction of the ACE2/Ang-(1–7)/Mas axis.

In this study, we evaluated the potential of oral and intranasal formulation of Ang-(1–7) to promote resolution of inflammation in a model of allergic lung inflammation. Our results clearly show that Ang-(1–7) interferes with the molecular pathways that regulate leukocyte survival and clearance, and promotes resolution of inflammation by driving apoptosis of eosinophils.

## Materials and Methods

### Animals

Male BALB/C mice from the Animal Facility of our Institution (Centro de Bioterismo—CEBIO, UFMG) were housed under a 12/12 h light-dark cycle (lights on at 06:00 h) with free access to standard chow and tap water. Mice (8–9 weeks of age, weighing 20–25 g) were randomly assigned to four groups: (i) saline-sensitized and saline-challenged, control group (CTRL; *n* = 15); (ii) ovalbumin (OVA)-sensitized and OVA-challenged group (OVA; *n* = 15); (iii) OVA-sensitized and OVA-challenged group treated by oral administration of Ang-(1–7)/hydroxypropyl β-cyclodextrin (HPβCD) [OVA + Ang-(1–7); *n* = 15]; and (iv) OVA-sensitized and OVA-challenged group treated by intranasal administration of Ang-(1–7)/HPβCD [OVA + Ang-(1–7); *n* = 10]. All experimental procedures were approved by the Ethics Committee for Animal Experimentation (CEUA) of the Federal University of Minas Gerais (UFMG), Brazil (Protocol# 309/2013).

### Induction of Asthma

Sensitization was made by two injections of OVA (100 µg per mouse, i.p.; Sigma, St. Louis, MO, USA) in 2% alum (aluminum hydroxide gel adjuvant; Brenntag) at 7-days interval (days 0 and 7). Challenge was made by 8 intranasal administration of 10 µg of OVA from days 12 to 19. CTRL group received PBS (0.2 mL/mouse, i.p.) and was challenged with PBS at the same time points.

### Ang-(1–7) Treatment

Inclusion of Ang-(1–7) into an oligosaccharide HPβCD cavity protects the peptide during its passage through the gastrointestinal tract (28). Therefore, in this study Ang-(1–7)/HPβCD inclusion compound was used to perform oral or nasal administration of Ang-(1–7), 24 h after the last OVA challenge. For oral administration, CTRL and OVA groups received vehicle (92 μg/Kg of HPβCD in distilled water; 100 µl, by gavage) and OVA+Ang-(1–7) group received Ang-(1–7)/HPβCD [60 μg/Kg of Ang-(1–7) and 92 μg/Kg of HPβCD in distilled water; 100 µl, by gavage]. For intranasal administration, mice received Ang-(1–7)/HPβCD [30 μg/Kg of the Ang-(1–7) and 46 μg/Kg of HPβCD in saline, 20 µl; OVA+Ang-(1–7) group] and CTRL and OVA groups received vehicle [46 μg/Kg of HPβCD in saline, 20 µl].

### Bronchoalveolar Lavage (BAL) and Lung Samples

Twenty-four hours after Ang-(1–7) treatment, mice were killed by anesthetic overdose and the trachea of each animal was exposed and cannulated with a polypropylene catheter (20 G). Airways were washed with 2 ml of ice-cold PBS. The trachea was then clamped and the lungs were collected in functional residual capacity. Left lung was collected for morphometric analysis and the right lung was removed, snap frozen in dry ice and kept at −80°C until assayed. Total number of leukocytes was counted in Neubauer chamber after staining with Turk’s solution. Differential leukocyte counts were performed on cytocentrifuge preparations (Shandon Cytospin III), stained with May–Grünwald–Giemsa and counted using oil immersion microscopy (×100 objective) using standard morphological criteria to identify cell types. Apoptosis of cells present in the BAL was assessed as described previously (8). Briefly, cells were cytocentrifuged, fixed and stained with May–Grunwald–Giemsa and counted using oil immersion microscopy (×100 objective) to determine the proportion of cells with distinctive apoptotic morphology (cell shrinkage, chromatin condensation, nuclear fragmentation, and disruption of cell membrane). At least 500 cells were counted per slide and the results expressed as percentage of cells with apoptotic morphology.

### Analysis by Immunofluorescence

Cells of BAL were centrifuged at 1.200 rpm for 5 min at 4°C, and the pellet was resuspended in PBS and total cell counts were performed. A total number of 5 × 10^5^ cells was taken to perform cytocentrifugation (Cytospin; Shandon Lipshaw Inc., PA, USA) in cell coverslips. Next, cells were fixed with 4% paraformaldehyde for 15 min and washed three times. Fc Block (CD16/32, BD Biosciences) was added for 30 min to block unspecific binding of antibody. To evaluate levels of NF-κB (P-p65) and caspase 3, cells were permeated for 30 min with Perm/Wash solution (1:12 in PBS–BSA 1%; BD Bioscience, USA) and incubated with antibody overnight. For Mas receptor, cells were incubated with primary antibody overnight. Next, cells were incubated with fluorescent secondary antibody (Alexa Fluor 488-Cell Signaling; 1:300; green). For extracellular staining, the antibody was added directly to cells. Negative controls were obtained by performing the assay in the absence of antibody. Finally, coverslips were mounted with Fluormount mounting medium (Sigma-Aldrich, USA) for analysis. Images were obtained in a Nikon Eclipse Ti microscope with laser confocal C2, equipped with three different lasers (excitation 405, 488, and 543 nm) and emission filter 450/50 nm (channel 1), 515/30 nm (channel 2), and 584/50 nm (channel 3). Fluorescence intensity was measured using a 6.3 Volocity software (Perkin-Elmer, USA), which calculates the mean of the fluorescence intensity (MFI) of around 30 cells for each animal. Antibodies used were as follows: Pp65-Alexa Fluor 488 (Cell Signaling; 1:100; green), the fluorescent nuclear marker propidium iodide (Cell Signaling; 1:1,000; red), Siglec-F (Santa Cruz; 1:100; blue), cleaved-caspase 3-Alexa Fluor 488 (Cell Signaling; 1:50; green), and Mas receptor (1:100; Alomone; green).

### Histological Analysis

Lung was prepared for histological analysis, as previously described (20). Peribronchial fibrosis was evaluated in lung sections (4 µm) stained with trichrome of Gömori (Trichrome Stain LG Solution; Sigma-Aldrich, USA). Digital images of airways obtained at 200× magnification were analyzed using the software Image-Pro Plus. Ten to twelve peribronchial areas per lung were outlined and quantified. Results are expressed as the percentage of extracellular matrix deposition area.

### Collagen I and III mRNA Expression

Real-time RT-PCR was performed to evaluate mRNA expression of collagen type I and III in the lung, as previously described (20). Briefly, total RNA from lung sample was extracted using TRIzol reagent (Invitrogen, San Diego, CA, USA), treated with DNAse (RNase-free; Invitrogen) and reverse transcribed with Moloney Murine Leukemia Virus Reverse Transcriptase (M-MLV RT; Promega, Madison, WI, USA). Endogenous GAPDH (internal control), collagen I and collagen III cDNA were amplified using specific primers (Table S1 in Supplementary Material) and SYBR green reagent (Applied Biosystems, CA, USA) in ViiA™ 7 System (Applied Biosystems, CA, USA). Relative comparative CT method was applied to compare gene expression levels between groups using the equation 2^−ΔΔCT^.

### Proteins Measured by Western Blotting

Samples of protein (50 µg) extracted from the lung (*n* = 4–5 from each group) were applied to polyacrylamide gel/SDS 10% and then transferred to nitrocellulose membranes. Membranes were incubated overnight with different primary antibodies: GATA3 (Rabbit anti-GATA3, 1:500, Abcam Labs, Cambridge, UK); total Ikβα (rabbit anti-t-IkBα; 1:1,000; Cell Signaling, MA, USA) or phosphorylated IkBα (rabbit anti-p-Ikβα; 1:500; Cell Signaling, MA, USA); total ERK1/2 (rabbit anti-t-ERK1/2; 1:1,000; Cell Signaling; MA, USA), or phosphorylated ERK1/2 (rabbit anti-p-ERK1/2, 1:500; Cell Signaling; MA, USA). Next, membranes were incubated with fluorescent secondary antibody [IRDye ^®^ 680 conjugated goat (polyclonal) anti-Rabbit IgG (H + L) diluted to 1:10,000 (Li-COR Biosciences, NE, USA)]. Levels were normalized to GAPDH levels in the same sample. Staining was visualized and quantified in a Li-COR Odyssey Scanner (Biosciences, NE, USA).

### Quantification of Eosinophil Accumulation in Lung

Pulmonary eosinophil peroxidase (EPO) activity was determined to estimate eosinophil recruitment to the lung parenchyma, as described elsewhere (29). Absorbance was read in an ELISA reader (Expert Plus ASYS Hitech GmbH, Eugenorf, Austria) at 492 nm. Values are expressed in optical density.

### Efferocitosys Assay *In Vivo*

Human polimorphonuclear cells (PMNs) were isolated from peripheral venous blood drawn from healthy volunteers (Ethics Committee of the Universidade Federal de Minas Gerais, Brazil—Institutional Review Board Project number 0319.0.203.000-11), after informed written consent, as described elsewhere (30). Briefly, PMNs were separated by gradient centrifugation over Histopaque-1119 and Histopaque-1077 (Sigma-Aldrich, USA) and plated at 5 × 10^6^ cells/well. Apoptosis (>80%) was induced by staurosporine (10 µM) by culturing the PMNs in complete RPMI 1,640 for 1 h at 37°C in 5% CO_2_ atmosphere. FVB/N mice were treated with Ang-(1–7) (3 μg/mice; ip), 30 min before PMNs apoptotic cells (4 × 10^6^ cells) were injected in animals bearing a 72 h peritonitis elicited by 0.1 mg of zymosan. Mice were killed after 3 h and exudates were collected for morphologic analysis of the percentage of macrophages containing apoptotic PMNs (31).

### Statistical Analysis

Results are expressed as mean ± SEM. Comparisons among three or more groups were performed by one-way ANOVA followed by Newman–Keuls *post hoc* test. Significance between two groups was assessed by Student *t*-test. All analysis and graphics were made with the GraphPad Prism software (version 5.0; La Jolla, CA, USA). The level of significance was set to *p* < 0.05.

## Results

### Treatment with Ang-(1–7) Decreased Eosinophil Accumulation

**Figure 1:**
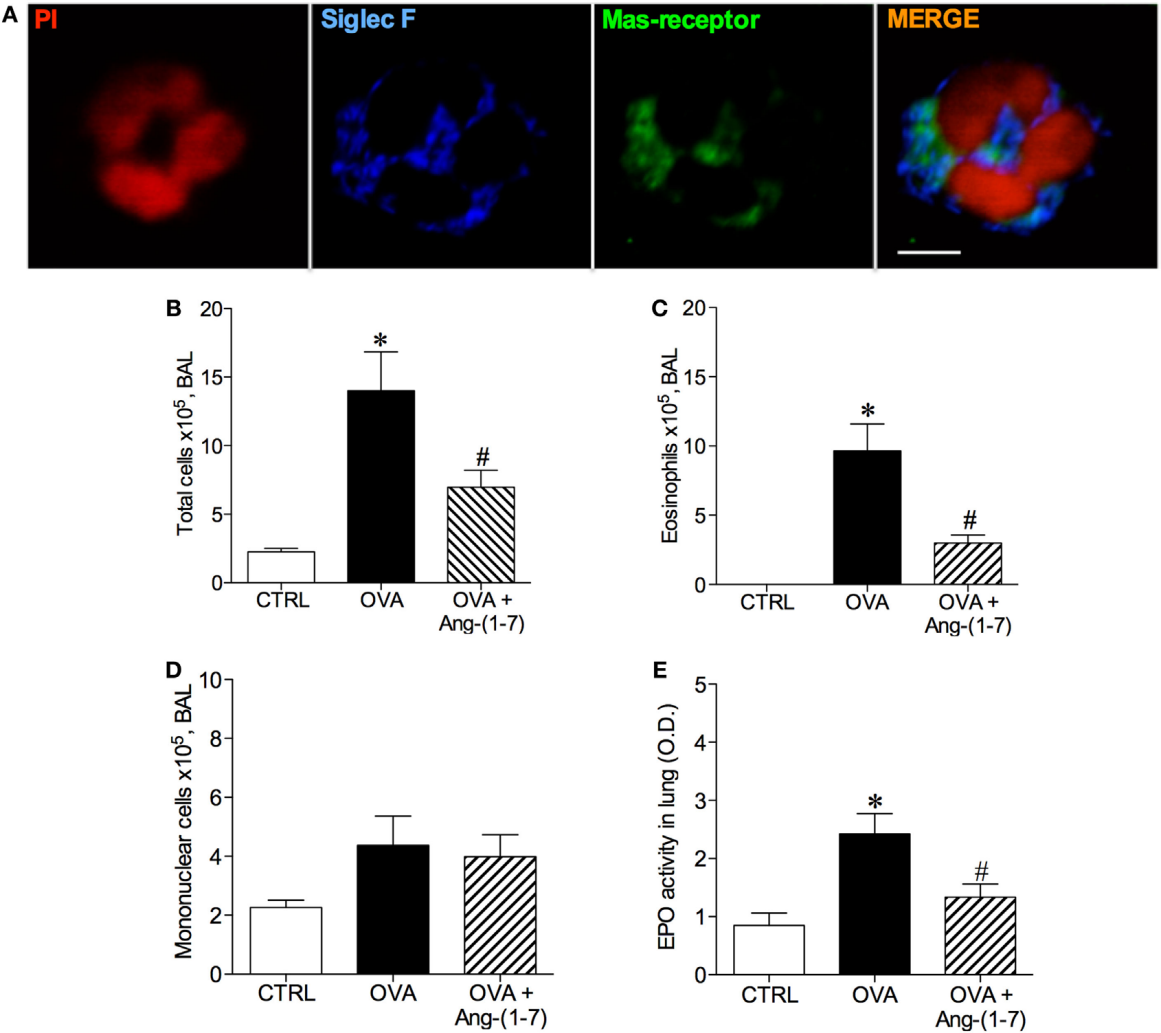
**(A)** Image of an eosinophil of the bronchoalveolar lavage (BAL) fluid illustrating Mas receptor expression by immunofluorescence; propidium iodide (PI), a marker of nucleus cell, in red; siglec F, a marker of eosinophil, in blue; and Mas receptor in green; Scale = 5 µm; **(B)** Number of total cells in the BAL; **(C)** Number of eosinophils in the BAL; **(D)** Number of mononuclear cell in the BAL; **(E)** Eosinophilic peroxidase (EPO) activity in the lung of control (CTRL), asthmatic (OVA) and asthmatic mice treated with oral administration of Ang-(1–7)/hydroxypropyl β-cyclodextrin (HPβCD) [60 µg/kg of Ang-(1–7) and 92 µg/kg of HPβCD]. Bars show mean ± SEM from five to six animals per group. *p ≤ 0.05 compared to CTRL and ^#^*p* ≤ 0.05 compared to OVA (one-way ANOVA followed by Newman–Keuls test).

Considering the majority of Ang-(1–7) actions described to date are mediated by Mas receptor (18, 19), using immunofluorescence, we first showed that Mas receptor is indeed present in both murine (Figure 1A) and human (Figure S1 in Supplementary Material) eosinophils.

Next, we treated mice with oral administration of the inclusion compound, Ang-(1–7)/HPβCD, 24 h after the last OVA challenge, a time point at which the number of eosinophils are maximal and neutrophils are close to baseline (8). As expected, challenge with OVA induced an increase in the number of total cells in BAL (Figure 1B) and eosinophils (Figure 1C). Ang-(1–7) significantly decreased the number of eosinophils in the BAL of OVA-challenged mice (3 × 10^5^ ± 1 vs 10 × 10^5^ ± 2 cells, in OVA group, Figure 1B), without altering the number of mononuclear cell (Figure 1D). Similar results were observed with intranasal administration Ang-(1–7) (Figure S2 in Supplementary Material). In keeping, oral Ang-(1–7) treatment also reduced the level of EPO activity in the lung (Figure 1E).

### Angiotensin-(1–7) Promoted Resolution of Inflammation by Inducing Eosinophil Apoptosis

**Figure 2:**
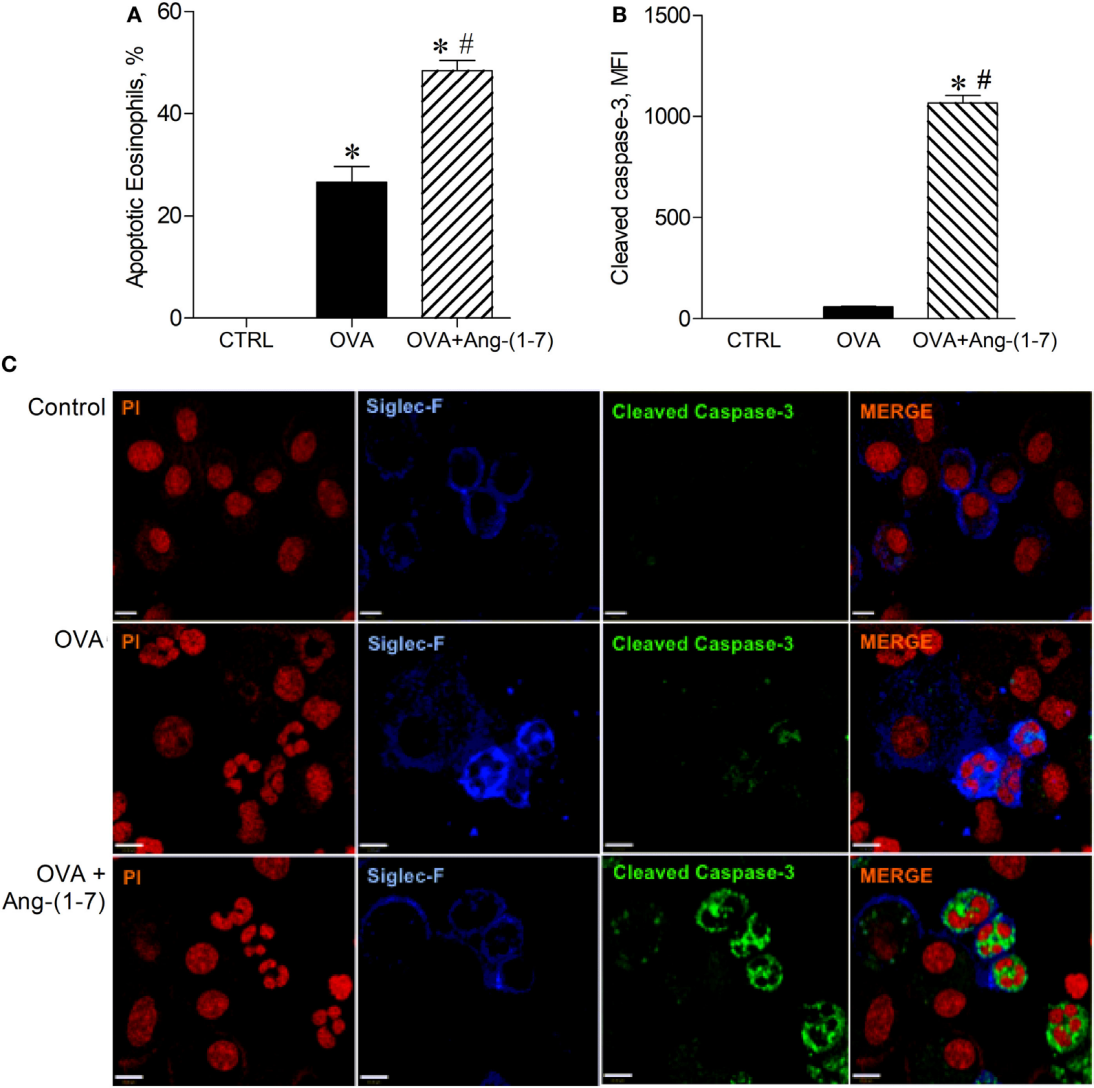
**(A)** Percentage of apoptotic eosinophils evaluated by morphological criteria in the bronchoalveolar lavage (BAL); **(B)** cleaved caspase 3 in eosinophils evaluated by immunofluorescence; **(C)** images illustrating cleaved caspase 3 immunofluorescence in eosinophils in the BAL; in control (CTRL), asthmatic (OVA), and asthmatic mice treated with oral administration of Ang-(1–7)/hydroxypropyl β-cyclodextrin (HPβCD) [60 µg/kg of Ang-(1–7) and 92 µg/kg of HPβCD]. Propidium iodide (PI), a marker of nucleus cell, in red; siglec F, a marker of eosinophil, in blue; and cleaved caspase 3 in green. MFI = mean fluorescence intensity. Scale = 43 µm. Bars show mean ± SEM from five to six animals per group. *p ≤ 0.05 compared to CTRL and ^#^*p* ≤ 0.05 compared to OVA (One-way ANOVA followed by Newman–Keuls test).

Next, we investigated whether oral administration of Ang-(1–7) could induce apoptosis of eosinophils, an important action to promote resolution of the inflammation. As shown in Figure 2A, Ang-(1–7) treatment increased the number of apoptotic eosinophils, as assessed by morphological criteria. Indeed, apoptosis of eosinophils was confirmed by a marked increase in caspase 3 expression in these cells (Figures 2B,C). This effect was also observed after intranasal administration of Ang-(1–7) (Figures S3A,B in Supplementary Material).

Following this observation, we investigated whether Ang-(1–7) could increase the ability of macrophages to engulf apoptotic leukocytes, a process referred as efferocytosis. Treatment with Ang-(1–7) induced a significant increase in efferocytosis of apoptotic PMNs cells (15.0 ± 1.4 vs 3.8 ± 1.2%, untreated mice).

### Angiotensin-(1–7) Promoted Resolution of Inflammation by Inhibition of the Survival of Eosinophils

**Figure 3:**
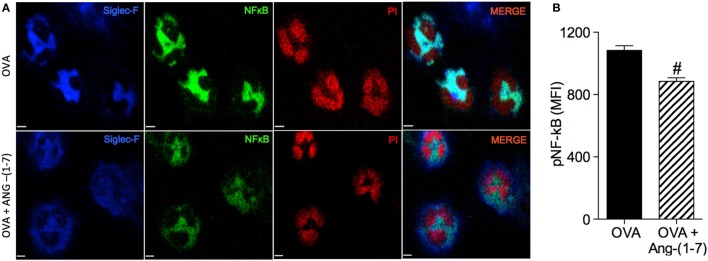
**(A)** Images showing phosphorylated NF-κB immunofluorescence in eosinophils in the bronchoalveolar lavage (BAL); **(B)** phosphorylated NF-κB in eosinophils evaluated by immunofluorescence; in asthmatic (OVA) and asthmatic mice treated with oral administration of Ang-(1–7)/hydroxypropyl β-cyclodextrin (HPβCD) [60 µg/kg of Ang-(1–7) and 92 µg/kg of HPβCD]. Propidium iodide (PI), a marker of nucleus cell, in red; siglec F, a marker of eosinophil, in blue; and p-p65 in green. MFI = mean fluorescence intensity. Scale = 15 µm. Bars show mean ± SEM from five to six animals per group. ^#^*p* ≤ 0.05 compared to asthmatic untreated mice (OVA; Student’s *t*-test).

Treatment with Ang-(1–7) decreased NF-κB staining in eosinophils of OVA-immunized and challenged mice (884 ± 23.3 vs 1,084 ± 29.2 MFI, OVA group; Figures 3A,B). This result points to one mechanism, importantly involved in the survival of leukocytes at the site of injury, by which Ang-(1–7) may resolve eosinophilic inflammation.

### Resolution of Allergic Lung Inflammation was Associated with Decreased Expression of ERK1/2, IκB-α, and GATA3 in the Lung

**Figure 4:**
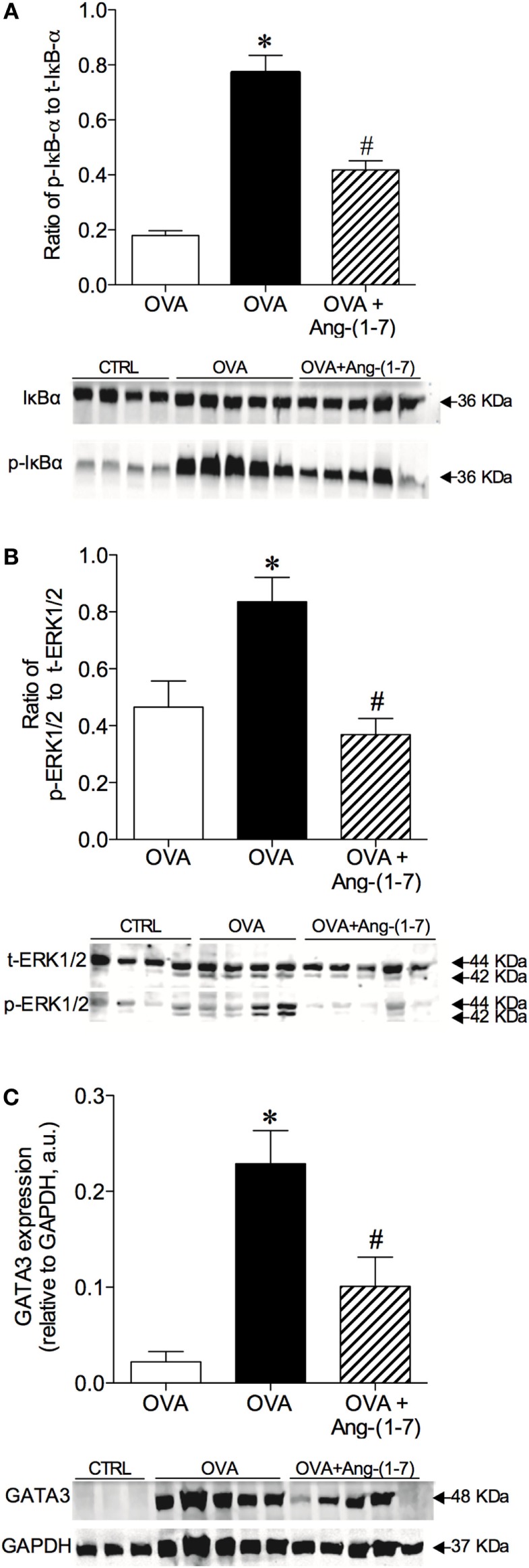
Densitometric quantification by Western blotting of intracellular signaling molecules of control (CTRL), asthmatic (OVA), and asthmatic mice treated with oral administration of Ang-(1–7)/hydroxypropyl β-cyclodextrin (HPβCD) [60 µg/kg of Ang-(1–7) and 92 µg/kg of HPβCD]. **(A)** Ratio of phosphorylated and total IκB-α; **(B)** ratio of phosphorylation and total ERK1/2; and **(C)** GATA3 expression in arbitrary units (a.u) in relation to GAPDH. Bars show mean ± SEM of 3–5 animals per group. Below each graph are representative blots illustrating molecular weight of each band (in KDa). *p ≤ 0.05 compared to CTRL; ^#^*p* ≤ 0.05 compared to OVA (One-way ANOVA followed by Newman–Keuls).

Oral treatment with Ang-(1–7) significantly attenuated the increase in the phosphorylation of IκB-α (0.418 ± 0.03; Figure 4A) observed in OVA-challenged mice (0.771 ± 0.06). No significant difference in total IκB-α was observed (data not shown).

Treatment with Ang-(1–7) greatly reduced the level of ERK1/2 phosphorylation in OVA-challenged animals (0.368 ± 0.05 vs 0.836 ± 0.08, OVA group; Figure 4B). No significant difference in total ERK1/2 was found (data not shown). Furthermore, oral treatment with Ang-(1–7) significantly reduced GATA3 expression in the lungs of OVA-challenged mice (Figure 4C). Similar results were observed with intranasal administration of Ang-(1–7) (Figures S4A–C in Supplementary Material). These data show that Ang-(1–7), given by oral or intranasal route, reduces IκB-α, ERK1/2, and GATA3 pathways, which are critical for immune cell migration, survival, differentiation, and the synthesis of pro-inflammatory mediators.

### Ang-(1–7) Decreased Expression of Early Markers of Fibrogenesis

**Figure 5:**
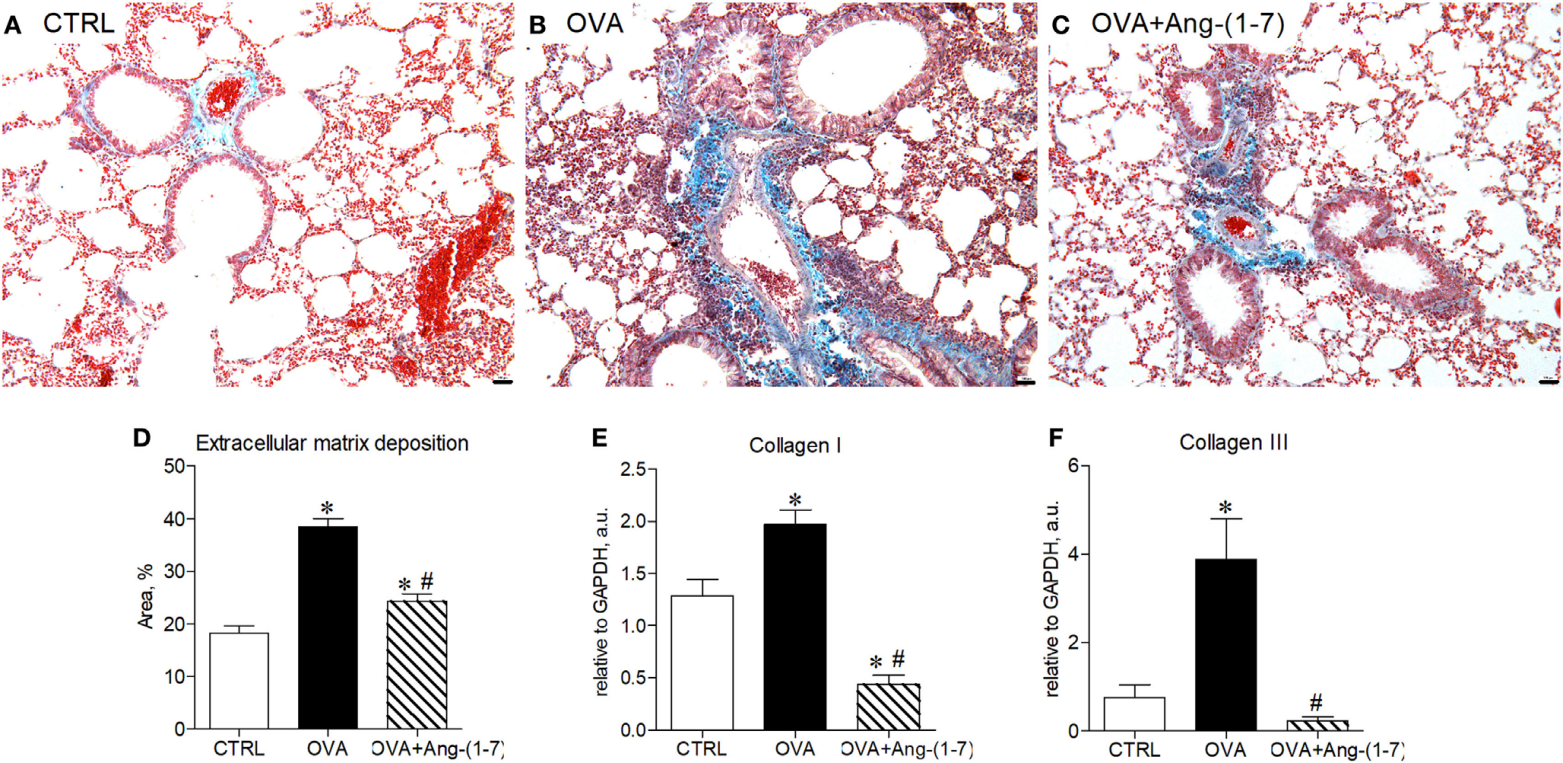
**(A–C)** Representative images of lung sections stained with trichrome of Gomori from control (CTRL), asthmatic (OVA), and OVA mice treated with oral administration of Ang-(1–7)/hydroxypropyl β-cyclodextrin (HPβCD) [60 µg/kg of Ang-(1–7) and 92 µg/kg of HPβCD]; Scale = 100 µm; **(D)** percentage of extracellular matrix deposition in the lung, evaluated by histology; **(E,F)** mRNA expression of collagen I and III in the lung. Bars show mean ± SEM from five to six animals per group. *p ≤ 0.05 compared to CTRL; ^#^*p* ≤ 0.05 compared to OVA (one-way ANOVA followed by Newman–Keuls test).

We have next assessed whether treatment with Ang-(1–7) could restore lung homeostasis. Figures 5A–C shows representative images of the lung showing asthma-induced alterations in extracellular matrix deposition. OVA-sensitized and challenged mice treated with oral administration of Ang-(1–7)/HPβCD had a marked reduction in extracellular matrix deposition in airway walls and alveolar parenchyma (Figures 5C,D). Furthermore, Ang-(1–7) treatment induced a marked decrease in collagen I (Figure 5E) and collagen III (Figure 5F) mRNA expression in the lung of OVA-challenged mice. Intranasal treatment with Ang-(1–7) also reduced extracellular matrix deposition in the lung of OVA-challenged animals (Figure S5 in Supplementary Material).

## Discussion

In this study, we evaluated the effect of Ang-(1–7) administration in the context of inflammatory resolution of allergic lung inflammation, a crucial event that prevents tissue damage and the consequent loss of organ functions (1, 4). We demonstrated that treatment with Ang-(1–7), at the peak of inflammation, i.e., after the influx of cells: (i) reduced accumulation of eosinophils in the BAL and in the lung, without altering the number of macrophages; (ii) induced apoptosis of eosinophils, as shown by increased number of apoptotic cells and increased caspase 3 expression in these cells; (iii) decreased NF-κB phosphorylation; (iv) decreased GATA3, ERK1/2, and IκB-α expression in the lung; and (v) decreased pulmonary remodeling. Furthermore, the presence of Mas receptor was shown, for the first time, in murine and human eosinophils and, Ang-(1–7) was able to induce efferocytosis of human PMNs. Altogether, these results demonstrate a pro-resolutive action of Ang-(1–7) in a model of eosinophilic inflammation.

Studies showed that activation of NF-κB is the major survival pathway of leukocytes, including eosinophils (32, 33). By contrast, inhibition of this pathway was associated with apoptosis of leukocytes and resolution of inflammation (32–34). In asthma, activation of NF-κB increases expression of genes encoding inflammatory cytokines and chemokines, maintaining the recruitment, activation, and survival of inflammatory cells (35, 36). The role of this transcription factor in the pathophysiology of asthma is supported by studies that showed that NF-κB-deficient mice develop only modest allergic lung inflammation (36). In addition, inhibition of NF-κB attenuates airway inflammation and pulmonary remodeling in a murine model of asthma (35, 36). Our data clearly showed that Ang-(1–7) decreases activation of NF-κB pathway in eosinophils and in the lung. This result shows that the pro-resolutive action of Ang-(1–7) in inducing leukocyte apoptosis and resolution of the allergic lung inflammation *in vivo* is, at least in part, regulated by the NF-κB phosphorylation.

Activation of a Th2 response during asthma is known to contribute to eosinophilic infiltration into the lungs, fibrosis, and loss of organ functions (7, 9). It has been demonstrated that GATA3 is an essential transcription factor for Th2 development and Th2-driven inflammation (37–39). Activation of ERK1/2 regulates GATA3 stability and Th2 differentiation (38). Treatment with Ang-(1–7) reduced GATA3 and ERK1/2 phosphorylation in the lung in OVA-challenged mice. In acute (35) or chronic asthma (20), decrease in ERK1/2 phosphorylation was associated with anti-inflammatory effect of Ang-(1–7). Therefore, resolution of inflammation induced by Ang-(1–7) was associated not only with a decrease in the number of eosinophils in the lung but also with a decrease in Th2 response at the development or at the chronic stages of the disease.

Increase in apoptosis of leukocytes and their clearance by macrophages are essential events to promote resolution of inflammation (1, 4). In a separated set of experiment, using a well-known approach to study efferocytosis *in vivo* (31), we showed that Ang-(1–7) treatment increased the clearance of the apoptotic cells by macrophages. This result adds an important criteria to establish Ang-(1–7) as an endogenous pro-resolving mediator.

Our results are also in keeping with previous studies that showed that anti-inflammatory, anti-proliferative, and anti-fibrotic effects of Ang-(1–7) are associated with inhibition of leukocyte migration and cytokine expression (19). Furthermore, Ang-(1–7) was shown to reduce key signaling pathways and molecules thought to be relevant for tissue remodeling (20, 23, 24, 35). In the present study, we provided strong evidence indicating that Ang-(1–7) can induce resolution of inflammation, through stimulation of caspase 3 and attenuation of NF-κB activation downstream mechanisms. Ang-(1–7) induced apoptosis of eosinophils without reducing the number of macrophages in the lung, this observation together with its ability to increase engulfment of apoptotic leukocytes, i.e., efferocytosis, it is important to be highlighted.

Our results showed that treatment with Ang-(1–7) in addition to resolving eosinophilic inflammation, had major physiological consequences in a model of asthma. Treatment with Ang-(1–7) diminished extracellular matrix accumulation and greatly reduced collagen I and III genes expression in the lung, which is of particular interest since collagen deposition in airways contributes to the lack of bronchial response in patients with chronic disease and it leads to severe unresponsive asthma.

Unregulated or prolonged inflammatory responses in the lungs can lead to tissue damage, pulmonary remodeling, and consequently compromise lung function (7, 40). Ang-(1–7), acting through the Mas receptor, has been shown to attenuate inflammation and fibrosis in different pathophysiological conditions (19). In previous study, treatment with Ang-(1–7) prevented pulmonary remodeling in a model of acute (35) or chronic asthma (20). Moreover, lack of the Mas receptor resulted in an intense degree of lung inflammation and remodeling in mice subjected to experimental model of chronic asthma (27). In present study, Ang-(1–7) induced resolution of inflammation via modulation of eosinophil apoptosis and the phagocytic clearance of apoptotic cells. These effects induced the return of pulmonary homeostasis through a decrease in extracellular matrix accumulation and a great reduction in collagen I and III genes expression in the lung.

Effects induced by Ang-(1–7) were observed either by oral or intranasal administration, which is important also to highlight. However, there are differences to be noted, intranasal dose (30 µg/kg) was half of oral dose (60 µg/kg), and complete resolution after intranasal administration was 24 h faster than after oral administration (data not shown). These information may favor intranasal administration, similar to standard treatments available. Nevertheless, asthma is not only a lung disease but it is also a systemic disease and correlates with less favorable respiratory function and response to standard treatment (41–44). Taking this into account and, the fact that Ang-(1–7) is an endogenous peptide already passed phase 1 clinical test, oral administration can present additional important advantages. In asthmatic patients, extra-pulmonary comorbidities contribute substantially to poor asthma control. Patients with difficult-to-control asthma also can present rhinitis, chronic rhinosinusitis, gastroesophageal reflux, obstructive sleep apnea, vocal cord dysfunction, obesity, dysfunctional breathing and anxiety/depression (45). Thus, the observation that Ang-(1–7) is effective after oral route can provide clinical benefits for treatment of allergic asthma, as it can be better tolerated than nebulization or than standard drugs, and it can act sistemically reducing overall inflammation and optimizing health of patients.

In conclusion, we unveil a novel action of the peptide Ang-(1–7), resolution of allergic lung inflammation. Our data demonstrated that treatment with Ang-(1–7) in mice with allergic lung inflammation fulfills all the criteria to be considered a novel resolution-inducing mediator. Therefore, together with previous findings showing that Ang-(1–7) presents an important anti-inflammatory effect, and considering that, it is an endogenous peptide already being subjected to phase 1 clinical trial, these data will accelerate the research efforts for the development of new Ang-(1–7)-based pharmacological strategies to control, prevent, and treat chronic inflammation-related diseases, such as asthma.

## Ethics Statement

All experimental procedures were approved by the Ethics Committee for Animal Experimentation (CEUA) of the Federal University of Minas Gerais (UFMG), Brazil (Protocol# 309/2013) and the Ethics Committee of the Universidade Federal de Minas Gerais, Brazil—Institutional Review Board Project number 0319.0.203.000-11.

## Author Contributions

GM and LCB: study conception and design, data acquisition, analysis and interpretation, and drafting and revising the manuscript; MR-M and LSB: data analysis and interpretation, and revising the manuscript; AR, JG, DM-S, AO, and DP: data acquisition, analysis, and interpretation; MT, VP, and RS: study conception and design, data analysis and interpretation, and manuscript edition and revision; MC-S: study conception and design, data analysis and interpretation, drafting, editing, and revising the manuscript. All authors approved the final version of manuscript.

## Conflict of Interest Statement

The authors declare that the research was conducted in the absence of any commercial or financial relationships that could be construed as a potential conflict of interest.
