# Supplementary material for: Angiotensin-(1–7) Promotes Resolution of Eosinophilic Inflammation in an Experimental Model of Asthma
Source: Front Immunol. 2018 Jan 29;9:58. doi: 10.3389/fimmu.2018.00058 (PMC5797293; doi:10.3389/fimmu.2018.00058)
Supplement: Supplementary file 1 [file Image_1.pdf]

# SUPLEMMENT

## ANGIOTENSIN-(1-7) PROMOTES RESOLUTION OF EOSINOPHILIC INFLAMMATION IN AN EXPERIMENTAL MODEL OF ASTHMA

Giselle S Magalhaes,[1*] Lívia C Barroso,[2*] Alessandra C Reis,[3] Maria Gloria Rodrigues-Machado,[1] Juliana F Gregório,[1] Daisy Motta-Santos,[1] Aline C Oliveira,[1] Denise A Perez,[3] Lucíola S Barcelos,[1] Mauro M Teixeira,[2] Robson A S Santos,[1] Vanessa Pinho,[3] Maria Jose Campagnole-Santos[1#]

[1]Department of Physiology and Biophysics, [2]Department of Biochemistry and Immunology and [3]Department of Morphology from the Biological Sciences Institute of the Federal University of Minas Gerais, Belo Horizonte, Brazil.

[*]These authors equally contributed to this work

**Running title:** Angiotensin-(1-7) a novel endogenous inflammation-resolving mediator

**#Corresponding author:** Maria Jose Campagnole-Santos

Department of Physiology and Biophysics;

Biological Sciences Institute

Federal University of Minas Gerais,

Av Antonio Carlos, 6627

31270-901, Belo Horizonte, MG, Brazil

mjcampagnole.ufmg@gmail.com

In this Supplement we present the following data: (*i*) immunofluorescence for Mas receptor in human eosinophils (Figure S1); (*ii)* data collected in Balb/C mice subjected to a model of asthma induced by ovalbumin (OVA) challenge and immunization treated (24h after last challenge) with intranasal Ang-(1-7)/HPβCD [30μg/kg of Ang-(1-7) included in 46μg/kg of HPβCD; Figures S2-S5]; (*iii*) images of unedited gels of experiments performed with oral (Figure S6-S8) or intranasal (Figures S5-S11) administration of Ang-(1-7). Intranasal or oral treatment was performed 24h after last challenge. Bronchoalveolar lavage and lungs were collected 24h after Ang-(1-7) administration.

## MATERIAL AND METHODS

**Mas staining in human eosinophils.** Human PMNs were isolated from peripheral venous blood drawn from healthy volunteers (Ethics Committee of the Federal University of Minas Gerais, Brazil - Institutional Review Board Project number 0319.0.203.000-11), after informed written consent, as described elsewhere (30). Briefly, PMNs were separated by gradient centrifugation over Histopaque-1119 and Histopaque-1077 (Sigma-Aldrich, USA). PMNs were centrifuged, at 1.200 rpm for 5 minutes at 4 °C, the pellet was resuspended in PBS, and total cell counts were made. From the total number were taken $5x10^5$ cells to perform cytocentrifugation (Cytospin; Shandon Lipshaw Inc., Pennsylvania, USA) in cells cover slips. Next, cells were fixed with 4% paraformaldehyde for 15 minutes and washed three times. Fc Block (CD16 / 32, BD Biosciences) was added for 30 minutes to block unspecific binding of antibody. Next, coverslips were permeated for 30 min with Perm/Wash solution (1:12 in PBS-BSA 1%; BD Bioscience, USA) and incubated with antibody overnight. Next, cells were incubated with fluorescent secondary antibody (Alexa Fluor 488-Cell Signaling; 1:300; green). Negative controls were obtained by performing the assay in the absence of antibody. Finally, coverslips were prepared with Fluormount (Aldrich Sigma, USA) for analysis. Images were obtained in a Nikon Eclipse Ti microscope with laser confocal C2, equipped with three different lasers (excitation 405, 488 and 543 nm) and emission filter 450/50 nm (channel 1), 515/30 nm (channel 2) and 584/50 nm (channel 3). Antibodies used were: MAS (Alomone, 1:100 green) and fluorescent nuclear marker PI (Cell signaling; 1:1000, red) diluted in permeabilization solution (Perm/Wash buffer; BD Biosciences, USA).

**TABLE S1.** Sequence of the primers used to perform qRT-PCR.

| Primer | Nucleotide sequence |
|---|---|
| Collagen 3 | FW GGTGGTTTTCAGTTCAGCTATGG<br>RV CTGGAAAGAAGTCTGAGGAATGC |
| Collagen 1 | FW CTTCACCTACAGCACCCTTGTG<br>RV GATGACTGTCTTGCCCCAAGTT |
| GAPDH | FW TGCGACTTCAACAGCAACTC<br>RV ATGTAGGCCATGAGGTCCAC |

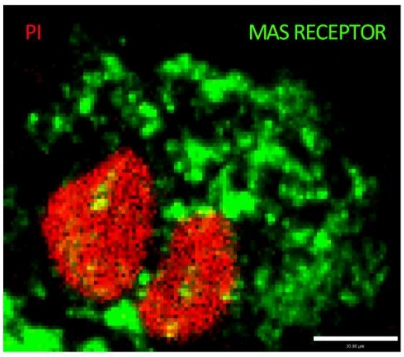


**Figure S1**. Confocal image illustrating Mas receptor immunofluorescence in a human eosinophil. Propidium iodide (PI), a nuclear fluorescent marker, in red; Mas receptor in green. Scale= 5μm

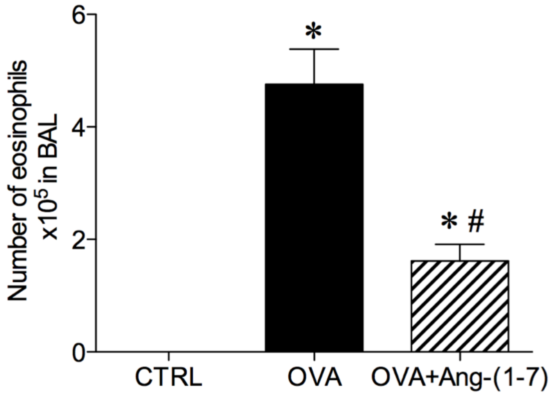


**Figure S2**. Number of eosinophils in the bronchoalveolar lavage (BAL) of Balb/C mice subjected to ovalbumin challenge and immunization (OVA) treated with intranasal Ang-(1-7)/HPβCD [30μg/kg of Ang-(1-7) included in 46μg/kg of HPβCD] or vehicle (empty HPβCD, 46μg/kg). As it can be seen, Ang-(1-7) treatment decreased eosinophil accumulation in the BAL. Bars show the mean ± SEM from 5-6 mice per group. *p≤ 0.05 in comparison to CTRL; #p≤ 0.05 in comparison to OVA (One-way ANOVA followed by Newman-Keuls test).

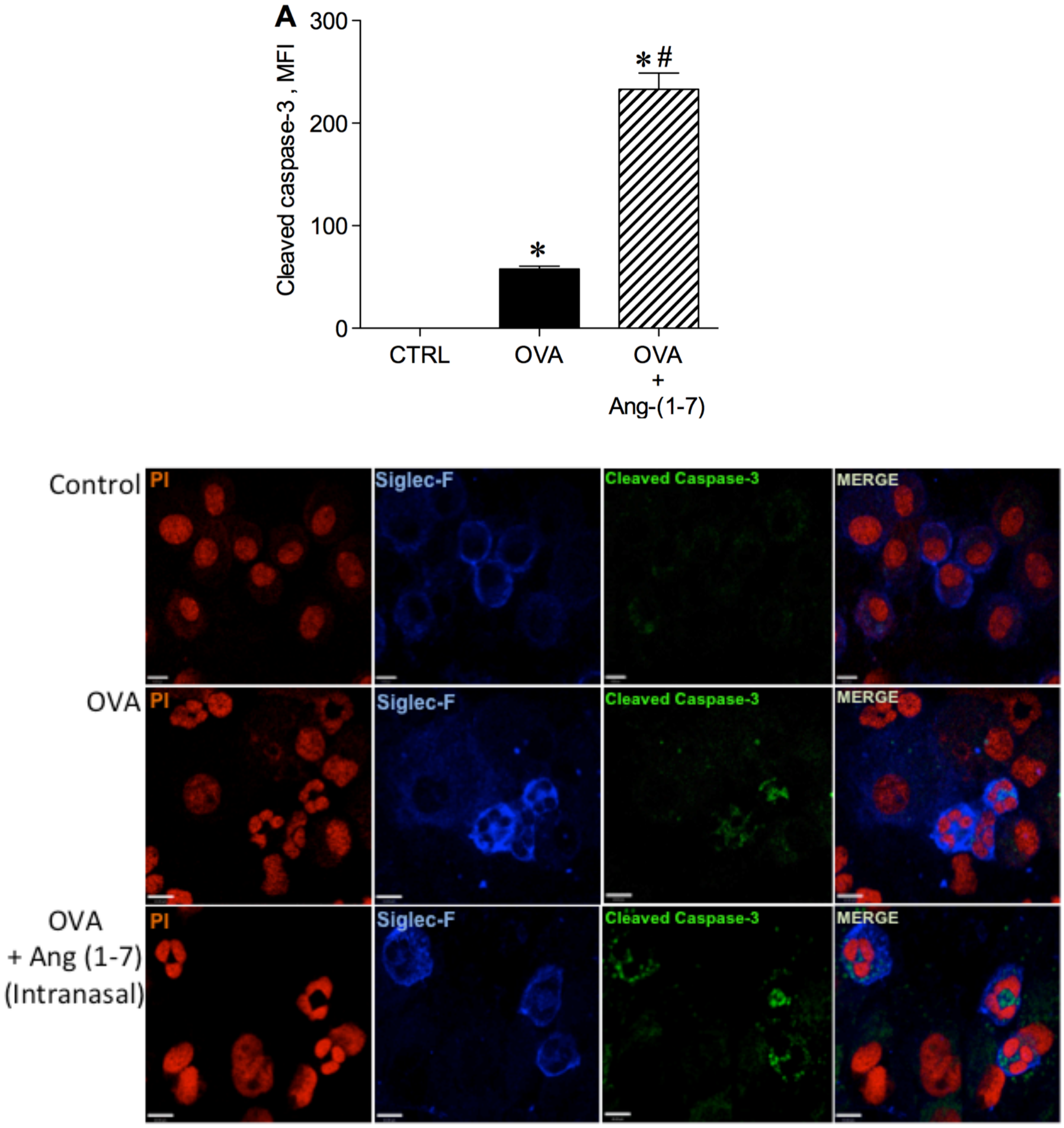


**Figure S3.** Level of cleaved-caspase-3 measured by immunofluorescence in the brochoalveolar lavage (BAL) of control (not asthmatic; CTRL), OVA sensitized and challenged (OVA) treated with vehicle (46µg/kg of HPβCD) and OVA mice treated with intranasal administration of Ang-(1-7)/HPβCD [30µg/kg of Ang-(1-7) included in 46µg/kg of HPβCD]. In (A), mean fluorescence intensity (MFI) of cleaved caspase-3. (B) Representative images of caspase-3 staining; nuclear fluorescent marker, propidium iodide (PI) in red; eosinophil marker, siglec F in blue; cleaved caspase-3 in green. Scale= 43 µm. As it can be seen, Ang-(1-7) increased caspase-3 levels indicating an increase in eosinophils apoptosis after intranasal administration. Bars show the mean ± SEM from 5-6 mice per group. *p≤ 0.05 in comparison to CTRL; #p≤ 0.05 in comparison to OVA (One-way ANOVA followed by Newman-Keuls test).

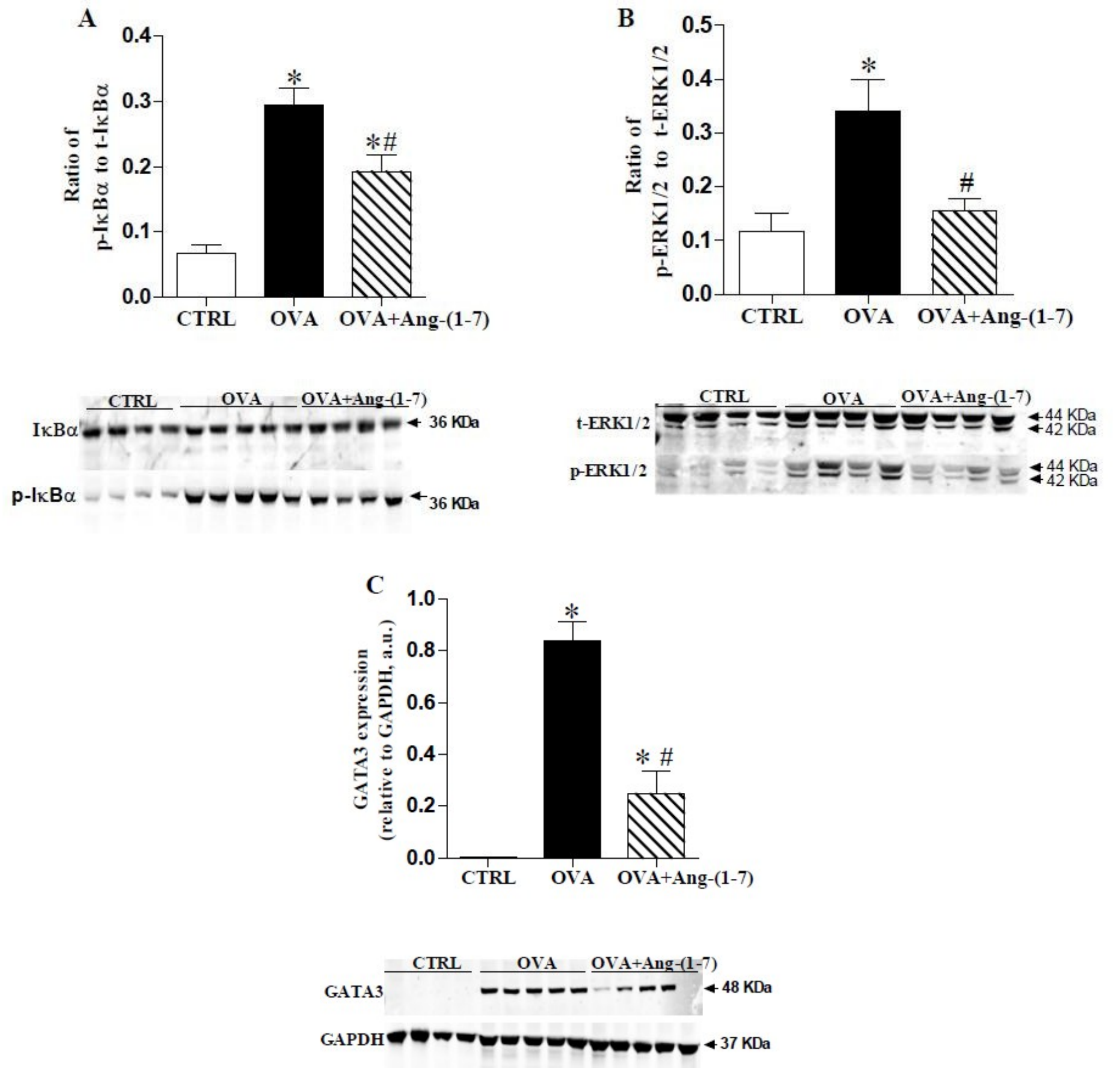


**Figure S4**. Level of phosphorylation of IκBα (A), ER1/2 (B) and GATA3 expression (C) in the lung of control (not asthmatic; CTRL), OVA sensitized and challenged treated with vehicle (OVA; 46μg/kg of HPβCD) and OVA mice treated with intranasal administration of Ang-(1-7)/HPβCD [30μg/kg of Ang-(1-7) included in 46μg/kg of HPβCD]. Bars show the mean ± SEM of densitometry quantification by Western blotting from 4-5 animals per group and bellow each graph are representative blots. As it can be seen, intranasal administration of Ang-(1-7)/HPβCD is associated with a decrease in signaling molecules crucial for the Th2 response. $*p \leq 0.05$ in comparison to CTRL; $\#p \leq 0.05$ in comparison to OVA (One-way ANOVA followed by Newman-Keuls test).

**A**

Control OVA OVA + Ang-(1-7)

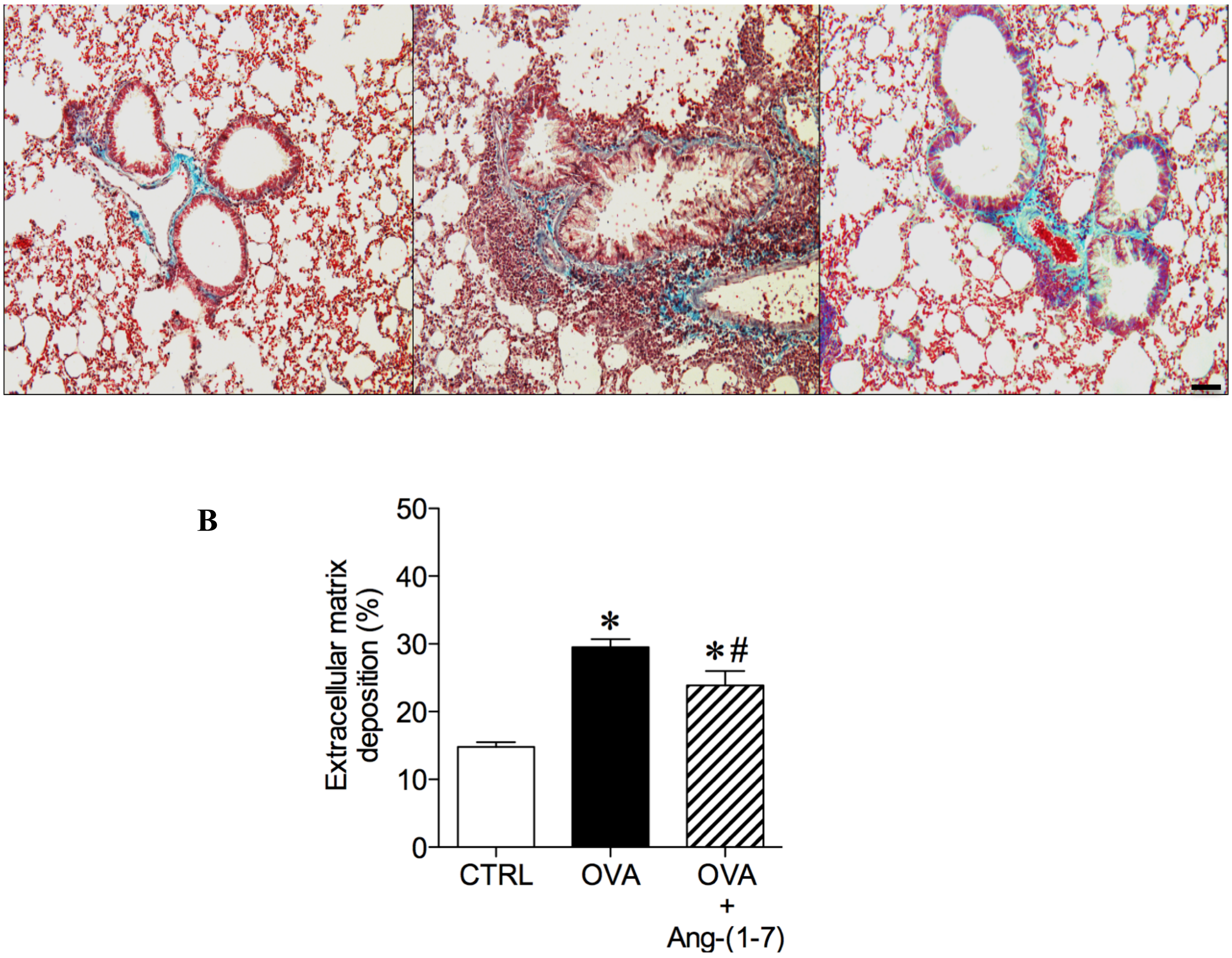


**Figure S5.** (A) Images of lung sections stained with trichrome of Gomori; (B) Extracellular matrix deposition in airway walls and alveolar parenchyma evaluated by percentage of the stained area of the lung of control (not asthmatic; CTRL), OVA sensitized and challenged treated with vehicle (OVA; 46µg/kg of HPβCD) and OVA mice treated with intranasal administration of Ang-(1-7)/HPβCD [30µg/kg of Ang-(1-7) included in 46µg/kg of HPβCD]. Scale 100 µm. Bars show the mean ± SEM from 4-5 mice per group. *p≤ 0.05 in comparison to CTRL; #p≤ 0.05 in comparison to OVA (One-way ANOVA followed by Newman-Keuls test).

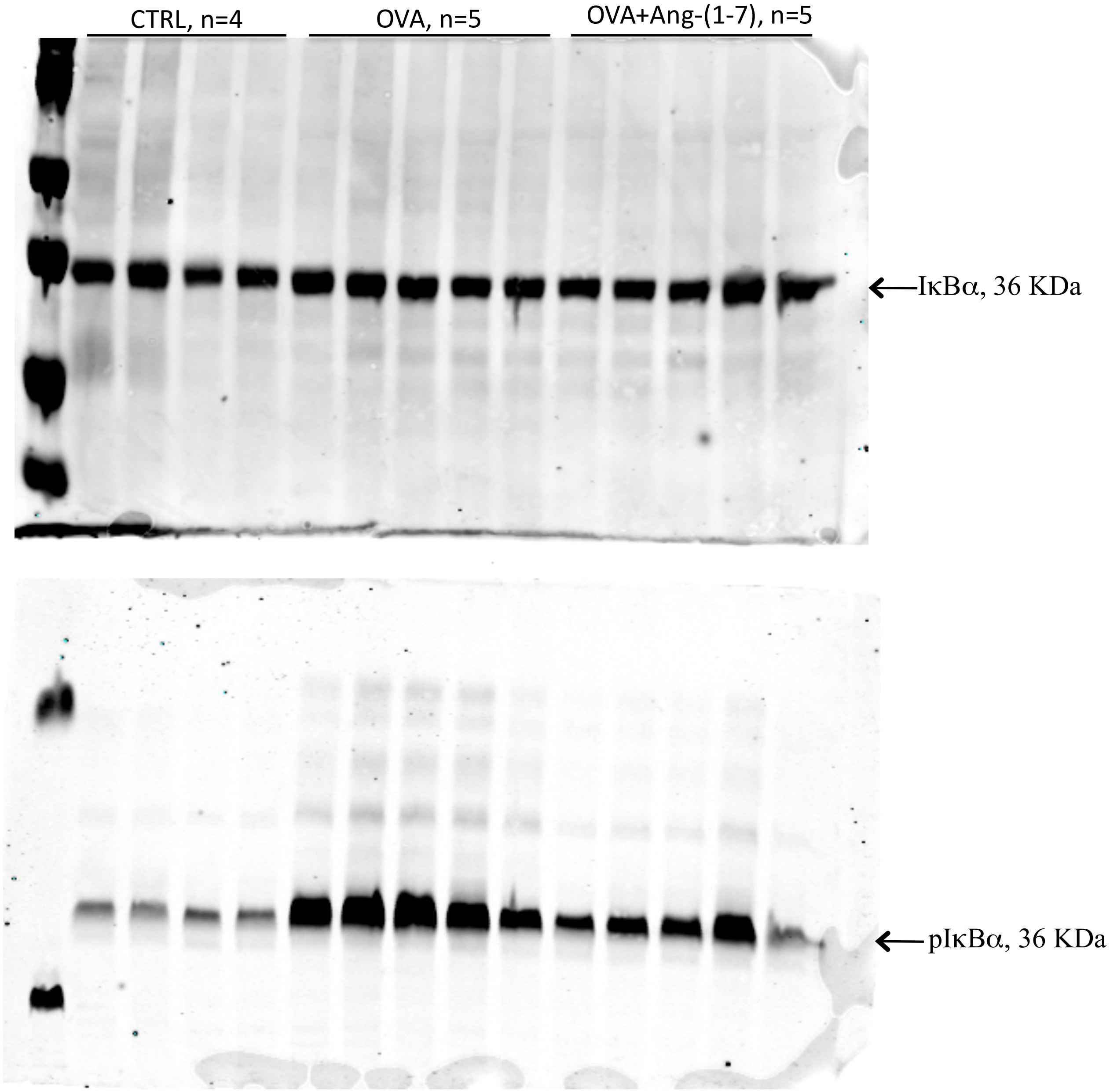


**Figure S6.** Unedited Western blotting gels showing bands for total (top gel) and phosphorylate (bottom gel) – IκBα of control (not asthmatic; CTRL), OVA sensitized and challenged treated with vehicle (OVA; 92 μg/kg of HPβCD) and OVA mice treated with oral administration of Ang-(1-7)/HPβCD [60 μg/kg of Ang-(1-7) included in 92 μg/kg of HPβCD].

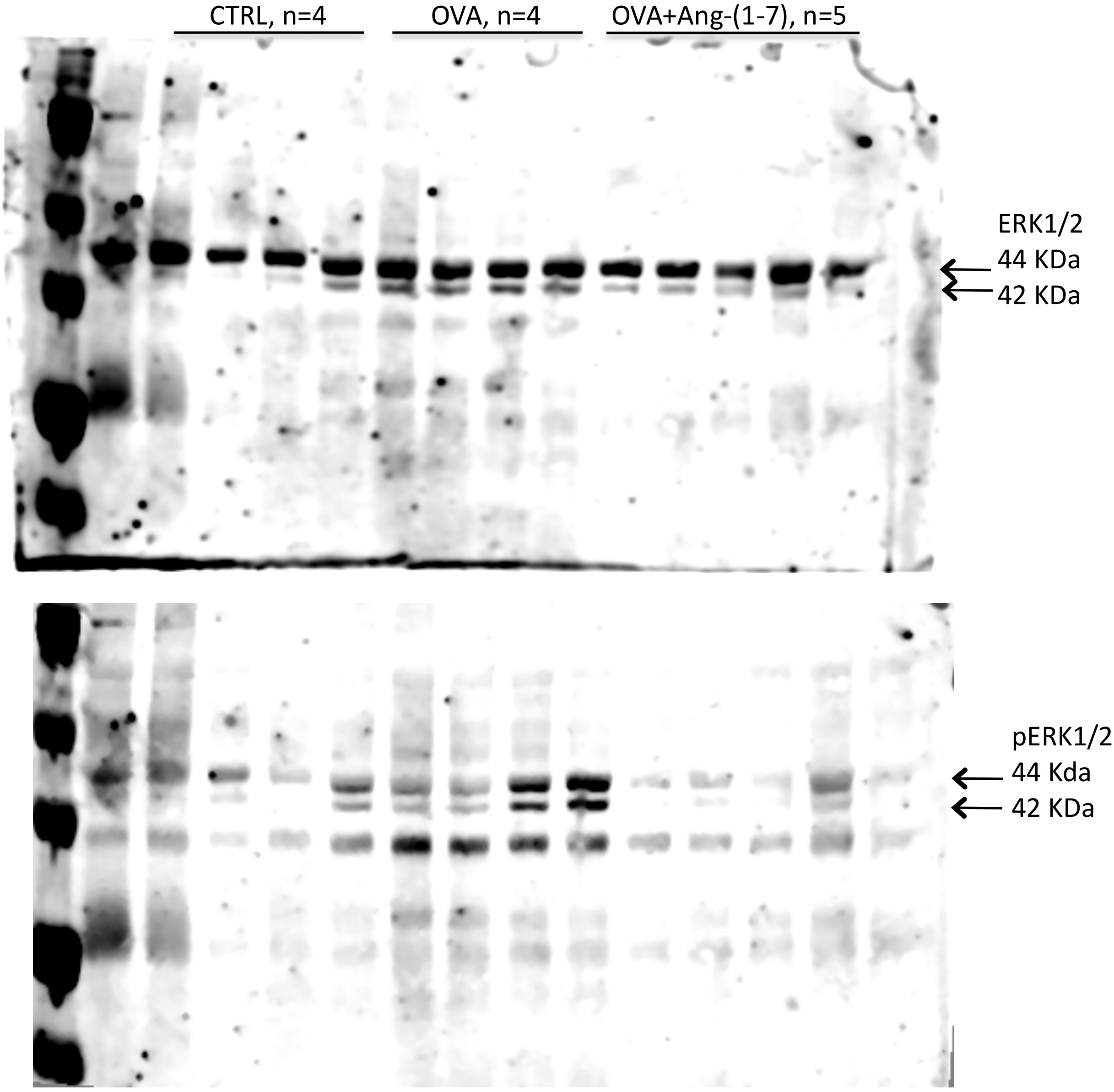


**Figure S7.** Unedited Western blotting gels showing bands for total (top gel) and phosphorylate (bottom gel) – ERK1/2 of control (not asthmatic; CTRL), OVA sensitized and challenged treated with vehicle (OVA; 92 µg/kg of HPβCD) and OVA mice treated with oral administration of Ang-(1-7)/HPβCD [60 µg/kg of Ang-(1-7) included in 92 µg/kg of HPβCD].

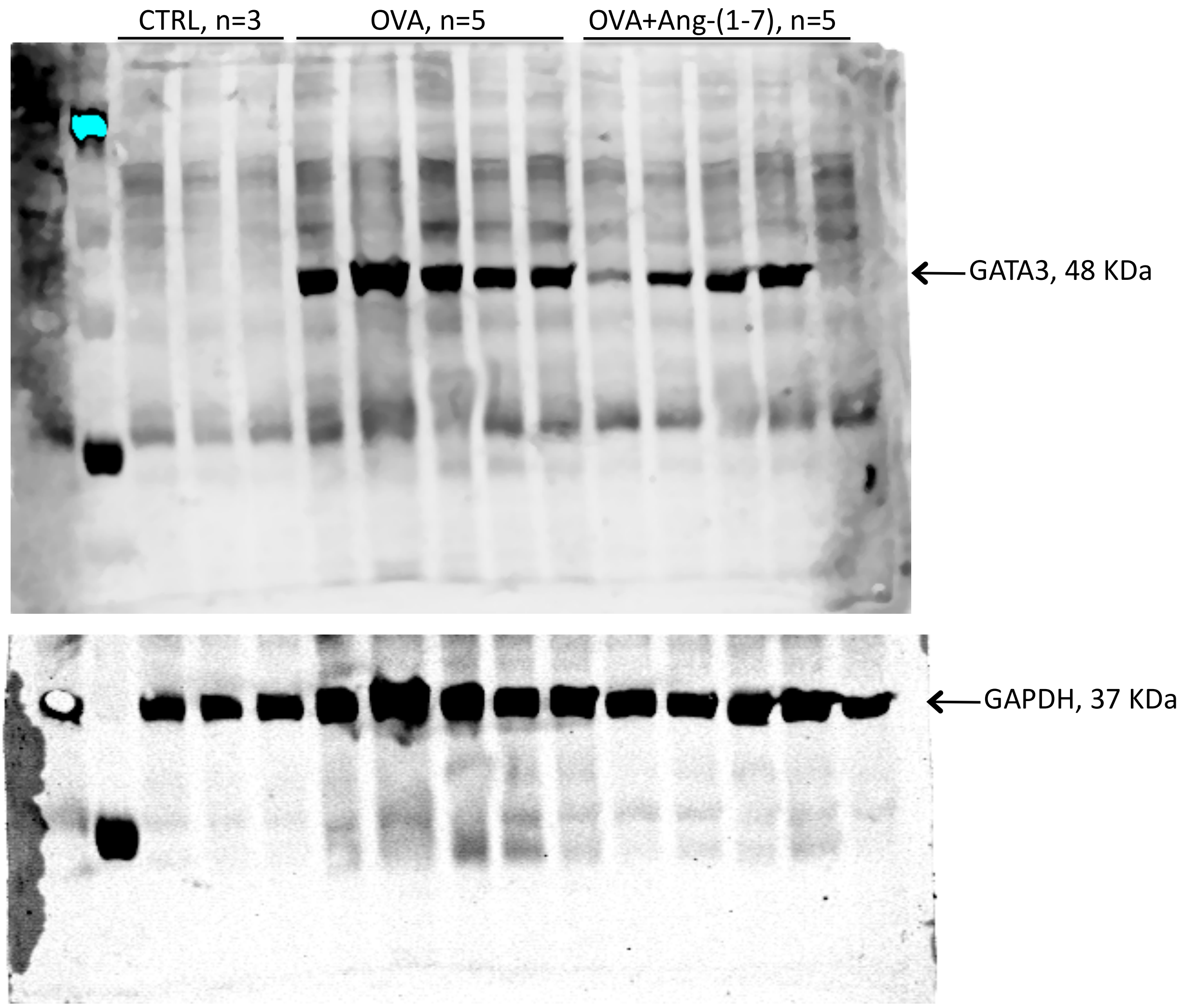


**Figure S8.** Unedited Western blotting gels showing bands for GATA3 (top gel) and GAPDH (bottom gel) expression of control (not asthmatic; CTRL), OVA sensitized and challenged treated with vehicle (OVA; 92 μg/kg of HPβCD) and OVA mice treated with oral administration of Ang-(1-7)/HPβCD [60 μg/kg of Ang-(1-7) included in 92 μg/kg of HPβCD].

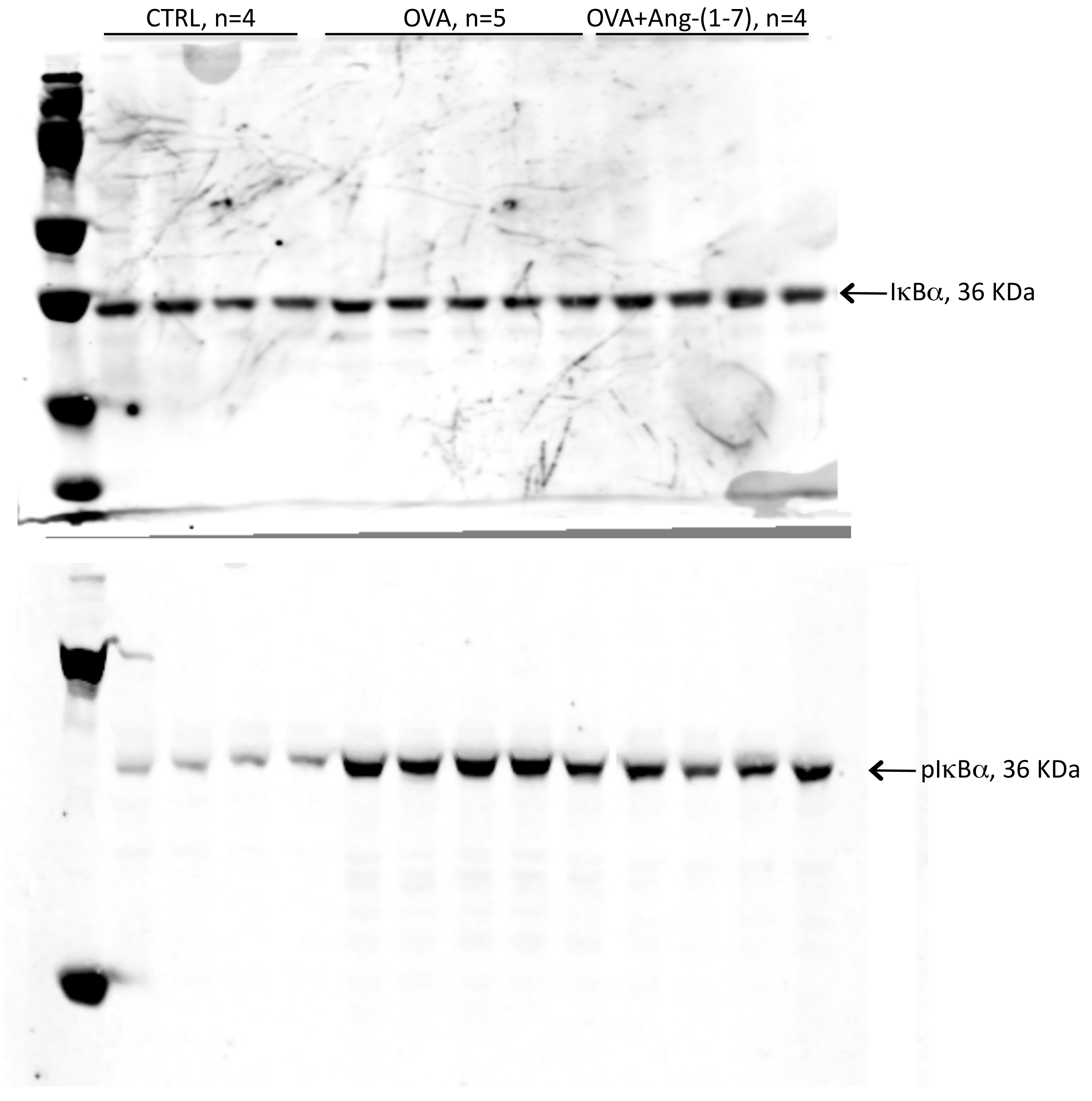


**Figure S9.** Unedited Western blotting gels showing bands for total (top gel) and phosphorylate (bottom gel) – IκBα of control (not asthmatic; CTRL), OVA sensitized and challenged treated with vehicle (OVA; 46μg/kg of HPβCD) and OVA mice treated with intranasal administration of Ang-(1-7)/HPβCD [30μg/kg of Ang-(1-7) included in 46μg/kg of HPβCD].

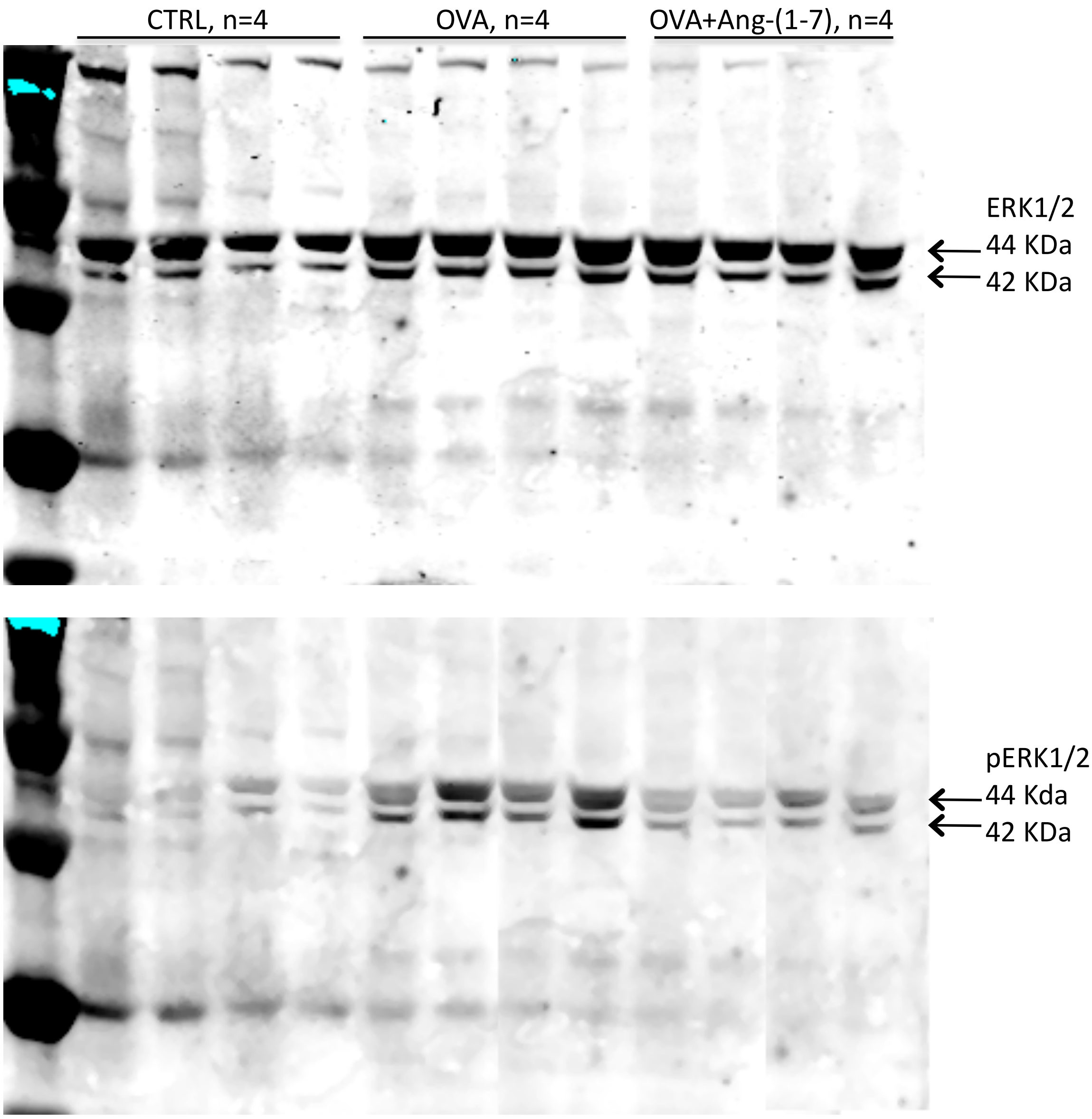


**Figure S10.** Unedited Western blotting gels showing bands for total (top gel) and phosphorylate (bottom gel) – ERK1/2 of control (not asthmatic; CTRL), OVA sensitized and challenged treated with vehicle (OVA; 46µg/kg of HPβCD) and OVA mice treated with intranasal administration of Ang-(1-7)/HPβCD [30µg/kg of Ang-(1-7) included in 46µg/kg of HPβCD].

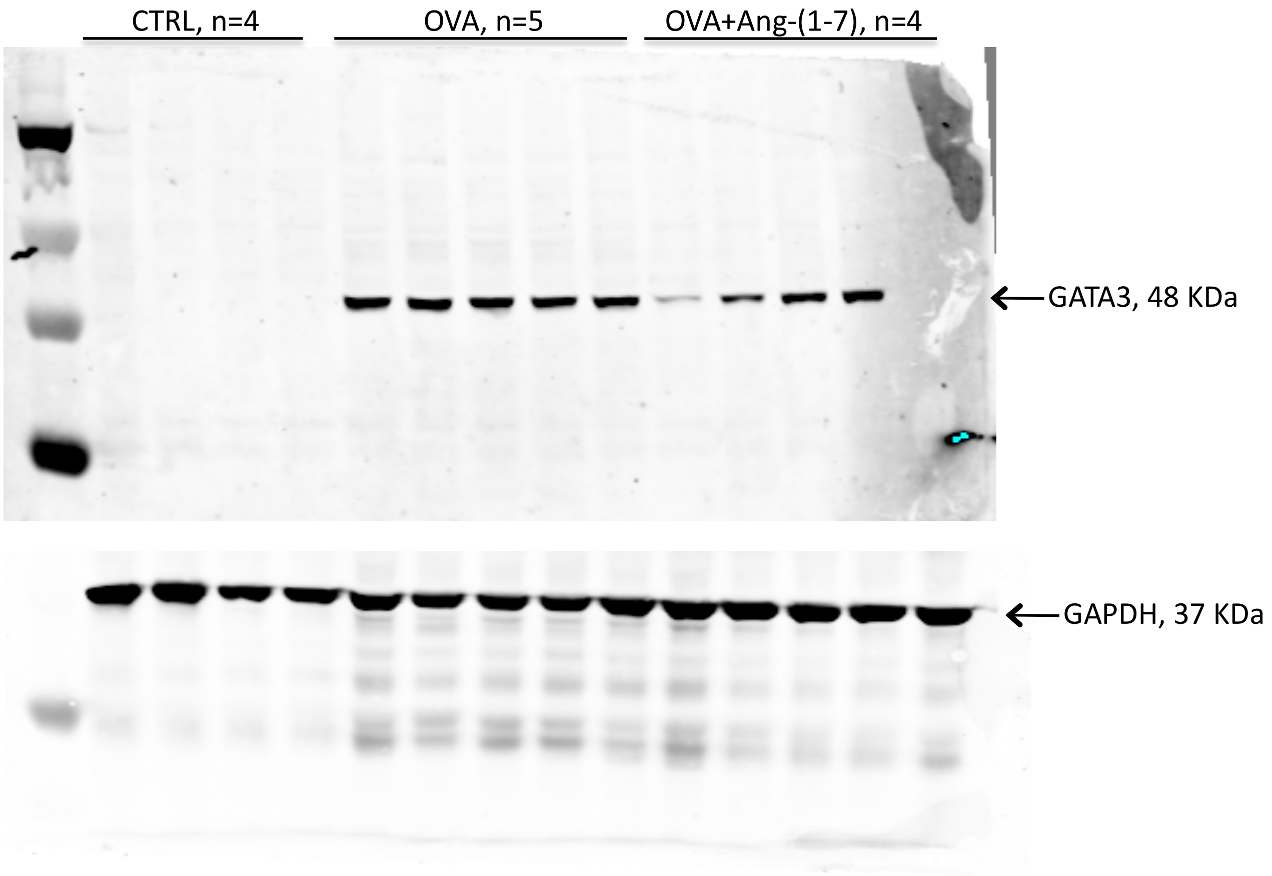


**Figure S11.** Unedited Western blotting gels showing bands for GATA3 (top gel) and GAPDH (bottom gel) expression of control (not asthmatic; CTRL), OVA sensitized and challenged treated with vehicle (OVA; 46μg/kg of HPβCD) and OVA mice treated with intranasal administration of Ang-(1-7)/HPβCD [30μg/kg of Ang-(1-7) included in 46μg/kg of HPβCD].
